# The different fates of two Asian horseshoe crab species with different dispersal abilities

**DOI:** 10.1111/eva.13271

**Published:** 2021-07-23

**Authors:** Qian Tang, Prashant Shingate, Yusli Wardiatno, Akbar John, Boon Hui Tay, Ywee Chieh Tay, Laura‐Marie Yap, Jasmin Lim, Hor Yee Tong, Karenne Tun, Byrappa Venkatesh, Frank E. Rheindt

**Affiliations:** ^1^ Department of Biological Sciences National University of Singapore Singapore City Singapore; ^2^ Institute of Molecular and Cell Biology A*STAR Biopolis Singapore City Singapore; ^3^ Environmental Research Centre IPB University Bogor Indonesia; ^4^ Institute of Oceanography and Maritime Studies (INOCEM) Kulliyyah of Science International Islamic University Malaysia (IIUM) Kuantan Pahang Malaysia; ^5^ Temasek Life Sciences Laboratory Singapore City Singapore; ^6^ School of Applied Sciences Republic Polytechnic Singapore City Singapore; ^7^ National Parks Board Singapore City Singapore

**Keywords:** benthic dispersal, climate change, conservation genomics, demographic reconstruction, seascape genomics, Sunda shelf

## Abstract

Impending anthropogenic climate change will severely impact coastal organisms at unprecedented speed. Knowledge on organisms’ evolutionary responses to past sea‐level fluctuations and estimation of their evolutionary potential is therefore indispensable in efforts to mitigate the effects of future climate change. We sampled tens of thousands of genomic markers of ~300 individuals in two of the four extant horseshoe crab species across the complex archipelagic Singapore Straits. *Carcinoscorpius rotundicauda* Latreille, a less mobile mangrove species, has finer population structure and lower genetic diversity compared with the dispersive deep‐sea *Tachypleus gigas* Müller. Even though the source populations of both species during the last glacial maximum exhibited comparable effective population sizes, the less dispersive *C*. *rotundicauda* seems to lose genetic diversity much more quickly because of population fragmentation. *Contra* previous studies’ results, we predict that the more commonly sighted *C*. *rotundicauda* faces a more uncertain conservation plight, with a continuing loss in evolutionary potential and higher vulnerability to future climate change. Our study provides important genomic baseline data for the redirection of conservation measures in the face of climate change and can be used as a blueprint for assessment and mitigation of the adverse effects of impending sea‐level rise in other systems.

## 1 INTRODUCTION

Global sea‐level change is one of the most prominent factors shaping coastal and marine natural communities through constant range shifts and changes in connectivity (Bird et al., 2007; Sheaves, 2009). Depending on life‐history parameters such as habitat preference and dispersal ability, coastal marine species differ in their evolutionary responses to environmental fluctuations in the wake of sea‐level change (Leprieur et al., 2016; Ludt & Rocha, 2015; Ni et al., 2014). An expanding genomic infrastructure and the recent development of powerful analytical approaches provide an opportunity to investigate the interplay between life‐history parameters and evolutionary responses (Gagnaire, 2020; Gagnaire et al., 2015). Understanding an organism's evolutionary responses to such range shifts is a critical prerequisite for the conservation of coastal marine biodiversity in this era of anthropogenic climate change (Chuang & Peterson, 2016; Miller et al., 2020; Nadeau & Urban, 2019). To evaluate species viability during environmental changes, most previous studies have estimated the evolutionary potential (e.g., genetic variation) of expanding populations by comparing them with populations in the core range (Berthouly‐Salazar et al., 2013; Braasch et al., 2019; Chen et al., 2018; Pierce et al., 2000; Robalo et al., 2020; Yang et al., 2016). However, many environmental fluctuations result in original habitats becoming uninhabitable, rendering core habitats difficult to identify, or individuals in core habitats undergo major demographic events (e.g., bottlenecks) which hamper a meaningful estimation of the evolutionary potential of expanding and receding populations. Two horseshoe crab species across the Singapore Straits in South‐East Asia, one of the world's most dynamic coastal landscapes, provide us with a unique opportunity to estimate the evolutionary potential of expanding coastal marine species whose source habitats during the last glacial maximum (LGM) remain unknown.

**FIGURE 1:**
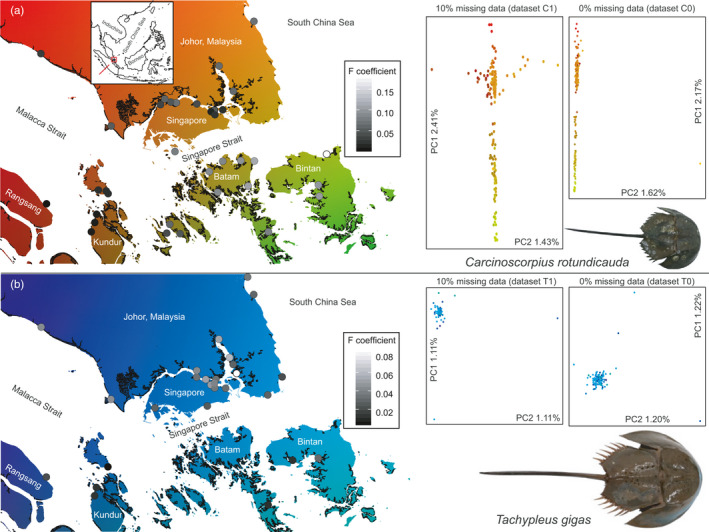
Genetic differentiation of two horseshoe crab species across the Singapore Straits. Sampling localities are illustrated as circles whose gray scale corresponds to locality‐specific *F* coefficients (calculated based on datasets C0 and T0). PCA plots are re‐scaled according to PC values, with the color of sample points adjusted to the color gradient on map. (a) *C*. *rotundicauda*; (b) *T*. *gigas*. Mangrove distribution by Giri et al. (2011) is illustrated as dark‐shaded coastal areas

Horseshoe crabs are long‐lived marine arthropods, often referred to as living fossils because of their nearly unchanged physical appearance over 455 million years (Rudkin et al., 2008). They occur across some of world's seas in four extant species. Two of them, *Carcinoscorpius rotundicauda* Latreille and *Tachypleus gigas* Müller, are widespread across coastal South‐East Asia, with highly overlapping ranges of distribution (John et al., 2018; Sekiguchi, 1988; Vestbo et al., 2018). The two species diversified 248 million years ago (Shingate et al., 2020) and possess different numbers of chromosomes (*C*. *rotundicauda* of 16 pairs and *T*. *gigas* of 14 pairs, Sekiguchi, 1988), which rules out potential for ongoing gene flow despite their highly overlapping ranges of distribution. Moreover, during artificial insemination experiments between the two species, eggs stopped development at blastula stage (Sekiguchi & Sugita, 1980). While similar morphologically, *C*. *rotundicauda* is relatively smaller (Figure 1), with a total length of ~30 cm (Srijaya et al., 2010), compared with *T*. *gigas*, which has a total length of ~40 cm (Tan et al., 2012). Their common names, mangrove horseshoe crab (*C*. *rotundicauda*) and coastal horseshoe crab (*T*. *gigas*), reflect habitat preference, with the former found in mangroves and mudflats and the latter on sandy beaches (Behera et al., 2015; Cartwright‐Taylor et al., 2011). Little information is available on the two species’ dispersal ability: one six‐month tracking study in Singapore indicates that *C*. *rotundicauda* is unlikely to travel across open sea (Cartwright‐Taylor & Ng, 2012), but allows for limited conclusions given that horseshoe crabs’ longevity exceeds 14 years (Sekiguchi, 1988). Based on preliminary genetic studies around the Malay Peninsula, population structure may be similar between *C*. *rotundicauda* and *T*. *gigas*, with the land barrier of the Malay Peninsula potentially impeding their dispersal (Adibah et al., 2015; Liew et al., 2015), but there is a lack of spatial analyses to explore dispersal patterns.

Although widespread across South‐East Asia today, the two horseshoe crab species may be limited in their contemporary ranges to few refugium habitats, as the drastic decline in effective population size during the inception of the last glacial period would suggest (Shingate, Ravi, Prasad, Tay, Garg et al., 2020). Akin to many other marine species (Crandall et al., 2012), horseshoe crabs repopulated the Sunda Shelf from unknown source populations at the end of LGM, as new coastal habitats were created by rising sea levels. Range expansions during the Holocene sea‐level rise may have boosted genetic variation as population sizes steadily increased (Wagner et al., 1997). However, such rapid expansion may also lead to a rapid loss of genetic diversity, as expanding populations become susceptible to environmental fluctuations (Eckert et al., 2008; Peter & Slatkin, 2013), especially in organisms with a slow reproductive rate such as horseshoe crabs (Shingate, Ravi, Prasad, Tay, Garg et al., 2020). Recent human‐induced habitat degradation, pollution, and overharvesting pose additional threats to the two horseshoe crab species (Carmichael et al., 2015; John et al., 2018, 2021). Even though the international conservation status remains ‘Data Deficient’ for both species, regional and national surveys have led to a listing of both species under various threat categories, for example ‘vulnerable’ for *C*. *rotundicauda* and ‘endangered’ for *T*. *gigas* in Singapore (Davison et al., 2008).

In this study, we screened hundreds of thousands of genome‐wide single nucleotide polymorphisms (SNPs) over 300 individuals of the two horseshoe crab species with a comprehensive geographic coverage across the Singapore Straits, a complex archipelagic nexus on the Sunda Shelf that has undergone dramatic habitat transformation during the Holocene (i.e., roughly the past ~10,000 years). We aimed to measure the dispersal ability and genetic diversity of the two horseshoe crab species as a novel type of baseline data for the implementation of conservation and mitigation measures against the effects of impending rising sea levels. The results of this research are already being implemented by some local authorities.

## 2 MATERIAL AND METHODS

Our sampling covered the Singapore Straits at a radius of 100 km around Singapore island (1°22’N, 103°48’E). A total of 188 *C*. *rotundicauda* and 116 *T*. *gigas* were collected across 46 locations (Figure 1; Table S1). All samples were captured alive and released after muscle tissue had been collected from one of their ambulatory legs, or blood had been drawn from the hinge, to ensure minimal intrusiveness. Tissue and blood samples were preserved in absolute ethanol and stored at −20°C for subsequent processing. We extracted genomic DNA using the DNeasy Blood & Tissue Kit (Qiagen). We followed a double‐digest RAD (ddRAD) library preparation protocol (Peterson et al., 2012) with few modifications. In brief, we used the restriction enzymes *EcoRI* and *MSeI* to digest the extracted genomic DNA and ligated the digested fragments with adapters. We pooled and selected ligated fragments of ~350 bp size using *Pippin Prep* (Sage Science) and performed eight PCR cycles on the size‐selected fragments for final library preparation before submission for next‐generation sequencing (Illumina HiSeq 4000, paired‐end 150 bp read length).

We checked sequence quality using *FastQC* (Babraham Bioinformatics) and demultiplexed all sequences using *process_radtag* as implemented in *Stacks* v2.4 (Rochette & Catchen, 2017). We aligned raw reads to whole‐genome sequences of *C*. *rotundicauda* (Shingate, Ravi, Prasad, Tay, Garg et al., 2020) (GenBank accession number VWRL00000000) and *T*. *gigas* (Shingate, Ravi, Prasad, Tay, & Venkatesh, 2020) (GenBank accession number JAALXS000000000), respectively, using *BWA*‐*MEM* as implemented in *BWA* v.0.7.15 (Li, 2013). Reads with MAPQ scores lower than 20 were discarded. Using *ref_map*.*pl*, as implemented in *Stacks* v2.4, we called 2,720,972 SNPs for *C*. *rotundicauda* (average stack depth 22.8x) and 2,779,046 SNPs for *T*. *gigas* (average stack depth 21.7x) without prior population assignment (per sample coverage weighted by sample size is 8.98–54.89 for *C*. *rotundicauda* and 7.85–31.33 for *T*. *gigas*). We did not assign individuals to populations based on their sampling locality because our sampling focused on maximizing spatial coverage to guarantee as much differentiation as possible in pairwise spatial distances for increased robustness of the IBD‐related analyses (see below), leaving uneven numbers of individuals at each site. Therefore, we excluded some subsequent analyses that require population assignment, for example, the calculation of private alleles per population. Moreover, as the area of study is relatively small compared with the mobility of horseshoe crabs, analyses based on sampling locality determined population assignment may not be informative. We used *PLINK* v1.9 (Purcell et al., 2007) to filter missing data. *PLINK* was also run to remove physically linked loci for some analyses but not for others (see below) (*indep*‐*pairwise* algorithm with a 25‐SNP window sliding 10 SNPs during each step; unphased‐hardcall *r* ^2^ threshold = 0.95).

To accommodate different requirements of downstream analytical programs, we generated six SNP datasets, three for each species: (C0) *C*. *rotundicauda* with 0% missing data and no linked loci (104,252 SNPs); (C0L) *C*. *rotundicauda* with 0% missing data while retaining linked loci (116,670 SNPs); (C1) *C*. *rotundicauda* with ~10% missing data and no linked loci (785,339 SNPs); (T0) *T*. *gigas* with 0% missing data and no linked loci (94,013 SNPs); (T0L) *T*. *gigas* with 0% missing data while retaining linked loci (108,728 SNPs); and (T1) *T*. *gigas* with ~10% missing data and no linked loci (432,544 SNPs). Detailed scripts outlining data quality control are appended in the supplement.

To examine genetic variation within individuals, within sampling localities, and among sampling localities for *C*. *rotundicauda* and *T*. *gigas* in our study area, we performed AMOVA, as implemented in the *R* package *poppr* (Kamvar et al., 2014), on datasets C0, C1, T0, and T1. We calculated observed and expected homozygosity as well as method‐of‐moments *F* coefficients for datasets C0, C1, T0, and T1 using the ‐‐*het* function available in *PLINK* v1.9. We averaged *F* for individuals collected at identical sampling localities to check spatial patterns in the distribution of genetic diversity. To obtain an alternative proxy of genetic diversity, we calculated effective population size based on genome‐wide linkage disequilibrium (LDNe), which is considered one of the superior ways of computing effective population sizes with single time point sampling (Waples, 2016). Moreover, as chromosomal linkage and recombination information are available for horseshoe crabs, we can rule out the usual shortcoming of the LDNe approach, such as the underestimation of recent effective population sizes (Waples et al., 2016). The estimation of contemporary LDNe was conducted on datasets C0L and T0L using *NeEstimator* v2.1 (Do et al., 2014), with a random mating model and minimum allele frequency >0.05. We computed fluctuations of LDNe over time on datasets C0L and T0L using *LinkNe* (Hollenbeck et al., 2016). *LinkNe* requires input of locus positions in centiMorgans (cM), requiring us to convert the unit of original locus positions from bp to cM using 10^6^ bp = 1 cM as an approximation based on the estimated genomic recombination rate of the Atlantic horseshoe crab (maternal 1.28 cM/Mb and paternal 0.76 cM/Mb) (Nossa et al., 2014). To guarantee the precision of LDNe estimation with *LinkNe*, we set bin sizes to 0.1 cM (over 10,000 pairwise comparisons in each bin) and the minimum allele frequency at >0.05. We used a generation time estimate of 14 years based on life‐history data (Sekiguchi, 1988) when converting the number of generations into years. As our study focuses on a relatively small geographical area (~100 km radius), relatedness can be a highly accurate proxy of genetic diversity. To estimate pairwise relatedness on datasets C0, C1, T0, and T1, we used a maximum‐likelihood algorithm as implemented in the *R* package *SNPRelate* (Zheng et al., 2012).

To visualize genomic diversification among individuals, we performed principal component analysis (PCA) based on the SNP genotype data, as implemented in *SNPRelate*, for datasets C0, C1, T0, and T1. In addition, we ran *ADMIXTURE* (Alexander et al., 2009), which uses maximum‐likelihood ancestry estimation, across datasets C0, C1, T0, and T1 to detect whether there are any population subdivisions within our study area. We performed discriminant analysis of principal components (DAPC, Jombart et al., 2010) on datasets C0 and T0 to provide a model‐free alternative for potential population subgrouping using the *R* package *poppr* (Kamvar et al., 2014). To verify whether the population genetic structure is contributed by genome‐wide differentiation or the ‘outlier’ loci under selection, we carried out two analyses based on the results of *PCA* and *ADMIXTURE* respectively. For the results of PCA, we calculated the correlations between the dominant principal component, PC1, and the SNP genotypes using function *snpgdsPCACorr*, as implemented in *SNPRelate*. For the results of *ADMIXTURE*, we ran a 20,000 bp sliding window moving in steps 5000 bp, performing *F*_st_ calculation for the nonadmixed individuals (with over 80% ancestry fraction) between probable ancestral populations using VCFtools v0.1.16 (Danecek et al., 2011).

To examine spatial‐genetic patterns, we calculated genetic distance matrices for datasets C0 and T0 using a relative dissimilarity approach (*diss*.*dist*) as implemented in the *R* package *poppr* (Kamvar et al., 2014) and geographic distance matrices using the least‐cost approach implemented in the *R* package *gdistance* (Etten, 2017) to account for landmasses between paired individuals. Subsequently, using *GenAlEx* v.6.51 (Peakall & Smouse, 2006), we modeled linear isolation by distance (IBD) and performed Mantel tests with 999 permutations to check for a spatial‐genetic correlation and correspondence. We also ran spatial autocorrelation analysis (Smouse & Peakall, 1999), as implemented in *GenAlEx* v6.51, for 999 permutations and 999 bootstraps, to examine dispersal patterns at different geographic distance classes. We mapped the spatial distribution of resistance to dispersal using *DResD* (Keis et al., 2013). *DResD* intakes the distance matrices of geographic and genetic distances to model nonlinear IBD, calculates IBD residuals of individual pairs, and maps the weighted means of IBD residuals to visualize the distribution of resistance to dispersal. We overlaid bathymetric data (SRTM15+) (Tozer et al., 2019) to verify whether sea depth is a barrier to the dispersal of the two horseshoe crab species.

## 3 RESULTS

**FIGURE 2:**
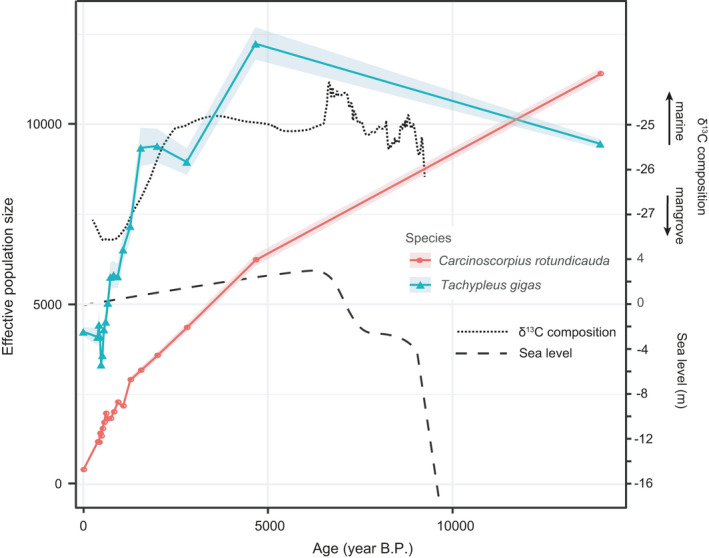
Effective population size across the Holocene of two horseshoe crab species. Effective population size is displayed with 95% confidence intervals (shaded area). Sea‐level fluctuations and carbon isotope composition in the Singapore Straits during the Holocene are based on Bird et al. (2010)

Overall, genetic variation is mostly contributed by within‐sample variation (~90%) for both horseshoe crab species across the Singapore Straits, while variation in individuals between sampling localities contributes ~2% for *C*. *rotundicauda* and 0% for *T*. *gigas* (Table S2). *C*. *rotundicauda* and *T*. *gigas* across the Singapore Straits are characterized by slight inbreeding, indicated by positive *F* coefficient values calculated for all four SNP datasets: (C0) 0.0529, (C1) 0.1003, (T0) 0.0324, and (T1) 0.0988. When applying equal regimes of missing data filtering (datasets C0 vs. T0, C1 vs. T1), *C*. *rotundicauda* has a lower genetic diversity, as indicated by higher *F* coefficients (Figure 1), than *T*. *gigas*. In both species, populations at the southwest end of the Singapore Straits exhibit the highest levels of genetic diversity (Figure 1), as shown by relatively low *F* coefficients when considering loci shared among all individuals (datasets C0 and T0). A relatively low genetic diversity in *C*. *rotundicauda* as compared to *T*. *gigas* is also indicated by pronounced differences in estimates of contemporary LDNe (age = 0), 395.8 in *C*. *rotundicauda* and 4237.6 in *T*. *gigas* (Figure 2), even though both species are estimated to have had a comparable LDNe at the end of the last glacial maximum (LGM) around 14,000 years ago (Figure 2). The LDNe of *C*. *rotundicauda* has continuously dropped throughout the Holocene whereas the effective population size of *T*. *gigas* has undergone a delayed but steeper drop over the last 5000 years, which roughly coincided with the recent rapid mangrove establishment across the Singapore Straits, following a period of more general stability in the early Holocene (Figure 2). We detected 45 pairs of related individuals (*r* < 0.0325, sharing an ancestor ≤5 generations ago) in *C*. *rotundicauda* but none in *T*. *gigas*. A total of 39 out of these 45 pairs were sampled at the southeast end of the Singapore Straits; 24 of them were collected at overlapping sites; and 19 of them at one particular pair of sites ~10 km from Bintan Island (Table S3).

**FIGURE 3:**
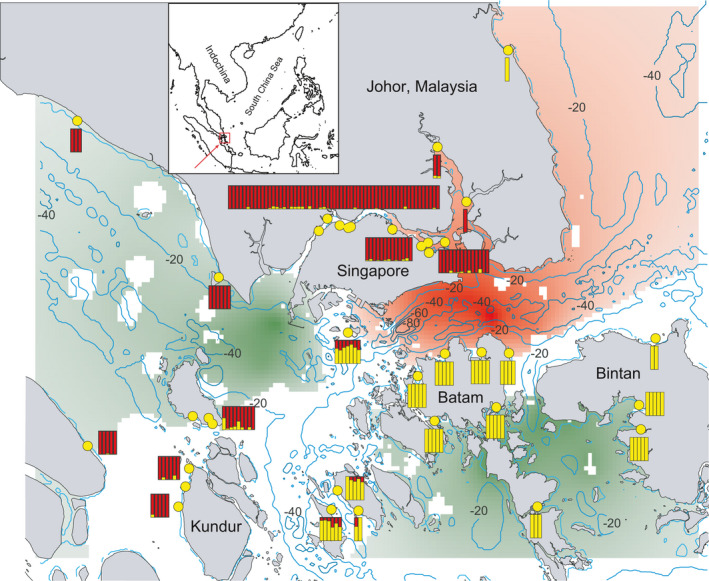
Map of resistance to *C*. *rotundicauda* dispersal across the Singapore Straits, generated using *DResD*. Red indicates areas of high resistance whereas green indicates areas of low resistance; sampling localities are indicated with yellow dots, bathymetry is illustrated with blue contour lines. *ADMIXTURE* bars (*K* = 2, dataset C0) are placed adjacent to correspondent sampling localities

Principal component analysis (PCA) failed to reveal major subdivisions within the two horseshoe crab species (Figure 1). Populations of *C*. *rotundicauda* are geographically arranged along a northwest–southeast genomic cline (Figure 1). In contrast, *T*. *gigas* emerges as virtually panmictic across the study area. Based on the cross‐validation error calculated for all tested *K* values (1–15) in the *ADMIXTURE* analyses (Table S4), the most likely number of ancestral populations is one for both species (i.e., *K* = 1). The second likely number of ancestral populations is two, with the second lowest cross‐validation error across all four datasets, for both species. When enforcing *K* = 2 (Figure S1), individuals of *T*. *gigas* display no geographic structure while individuals of *C*. *rotundicauda* are divided into two subgroups by the Singapore Straits, with intermediate individuals on Pulau Semakau (Figure 3), a small island in the middle of the Singapore Straits ~7 km south of Singapore. Based on the Bayesian information criterion, DAPC detected a single population for *C*. *rotundicauda* as well as for *T*. *gigas* (Figure S2). Even though *C*. *rotundicauda* displayed a spatially structured regional population across the Singapore Straits, the genetic divergence is shallow and does not associate with specific genotypes or regions across the genome (Figures S3 and S4). The PCA and *ADMIXTURE* results also suggest the population genetic pattern of spatial divergence in *C*. *rotundicauda* is most likely the result of isolation by distance rather than admixture of two distinct populations or natural selection. Otherwise, we would have observed genomic regions or genotypes that are highly associated with the spatial divergence.

**FIGURE 4:**
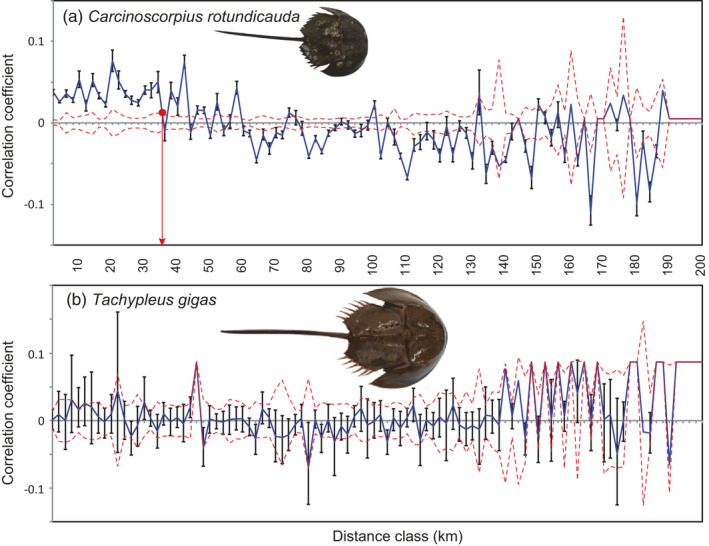
Spatial genetic autocorrelation in the two horseshoe crab species across the Singapore Straits. The ending point of a distance class is on the x‐axis, and the spatial autocorrelation coefficient (*r*) of genotypes in *C*. *rotundicauda* (a) and *T*. *gigas* (b) is on the y‐axis. The two dashed lines along the x‐axis are the permutated 95% confidence interval (CI) of autocorrelations under the null hypothesis of a random distribution of genotypes in space. Vertical lines are the bootstrapped 95% CIs with the mean genetic autocorrelation

Both horseshoe crab species display positive linear isolation by distance (IBD) in the study area, *C*. *rotundicauda* significantly so (*p* = 0.001, *R* ^2^ = 0.217) but *T*. *gigas* not (*p* = 0.32, *R* ^2^ = 0.0006). Results from spatial autocorrelation analyses (Figure 4) indicate that *C*. *rotundicauda* has a genetic patch size of ~35 km. In *T*. *gigas*, autocorrelation coefficients at initial distance classes do not significantly deviate from the null model, indicating that this species routinely disperses beyond the range of our study area (>200 km). In *C*. *rotundicauda*, *DResD* analysis detects significantly high resistance to dispersal around the Singapore Deeps (Bird et al., 2006) between Singapore and Batam Island around the deepest areas of sea of the entire study area (Figure 3). In *T*. *gigas*, relatively high resistance values emerge in a plain of shallow sea southwest of Singapore but with a lack of statistical power to support the robustness of these results (Figure S5).

## 4 DISCUSSION

We detected a relatively lower genetic diversity and finer population structure in the less dispersive, depth‐limited, mangrove‐adapted *C*. *rotundicauda* as compared to the more dispersive, open sea‐adapted *T*. *gigas*. Based on our study, we predict further steep declines in the genetic diversity of local *C*. *rotundicauda* with the impending human‐caused sea‐level rise. Further significant losses of genetic diversity directly translate into losses in evolutionary potential (Frankham et al., 2014; Palstra & Ruzzante, 2008), which will contribute to an unpredictable fate of *C*. *rotundicauda* populations in the Singapore Straits during the periods of intense environmental change expected for the coming decades and centuries. In contrast, the genetic diversity of *T*. *gigas* may be maintained or even be favored by a rapid sea‐level rise of up to 0.7 m (Stocker et al., 2013), but precise responses will critically depend on habitat availability and human disturbance.

Our population genetic analyses with a comprehensive geographic coverage yield the first firm evidence on differences in dispersal ability between the two horseshoe crab species (Figure 4), particularly as regards the depth‐limited dispersal of *C*. *rotundicauda* (Figure 3). Our approach provides an analytical framework for the study of movement ecology and life history where conventional marking and tracking methods are not as cost‐effective, especially in long‐lived species such as horseshoe crabs whose benthic life remains cryptic (Rudloe, 1979; Sekiguchi, 1988). Given size is important for the dispersal distance of active dispersers (Jenkins et al., 2007), our findings on the more limited dispersal in the smaller *C*. *rotundicauda* as compared to the larger *T*. *gigas* suggest that active benthic dispersal of adults, rather than passive phytoplanktonic dispersal of larvae, better explains the differences in dispersal distances of the two species. This is consistent with observations of limited larval dispersal in the other two horseshoe crab species from the Atlantic and East Asia (Botton & Loveland, 2003; King et al., 2005; Pierce et al., 2000; Yang et al., 2009).

Our sampling of horseshoe crabs from randomly mixed cohorts may underestimate LDNe (Waples et al., 2014). Given that the two horseshoe crab species have a similar lifespan and generation time, we expect the same level of bias in LDNe for both species, ruling out systemic biases in our results. Our results indicate that the more dispersive *T*. *gigas* has lost almost half of the genetic diversity during the expansion from the source population whereas the less dispersive *C*. *rotundicauda* has lost more than 95% (Figure 2). Loss of genetic diversity, as reflected in reduced effective population size, may be the consequence of sequential founder effects (Clegg et al., 2002), which both species would have needed to undergo in order to re‐expand across the vast shelf. However, differences in the degree of diversity loss between the two species may correspond to their different dispersal abilities. *T*. *gigas* was able to maintain genetic exchange across fairly distant populations, keeping diversity loss in check (Berthouly‐Salazar et al., 2013), while *C*. *rotundicauda* branched out into increasingly fragmented and isolated populations across the Sunda Shelf. Besides differences in dispersal ability, differences in their ability to adapt to novel coastal habitats created by rapid sea‐level rise may also have contributed to the difference in genetic diversity. However, this can be only tested with exclusive sampling beyond our study area to verify whether populations of *C*. *rotundicauda* from across the Sunda Shelf display the same level of low genetic diversity.

Our results provide important baseline data for the conservation of horseshoe crabs in the Singapore Straits and for mitigation against the future effects of climate change. These baseline data, which allow insights into both genetic diversity and dispersal ability, illustrate how dispersal ability may have contributed to present‐day patterns of genetic diversity and therefore may offer superior conservation recommendations as compared to the conventional baseline data of census population size or effective population size alone. The gradual and continuous drop of effective population size in *C*. *rotundicauda* over the last 10,000 years stands in stark contrast to the massive expansion and coastal domination of its preferred mangrove habitat in the region (Bird et al., 2010). This seeming contradiction underscores the importance of including a species’ dispersal ability and evolutionary trajectory in conservation planning (Hoban et al., 2013; Laikre, 2010). Our dataset affords surprising small‐scale resolution by capturing ongoing sequential founder effects of *C*. *rotundicauda* populations on Batam and Bintan islands to the south of the Singapore Straits. Individuals sampled around the latter two islands exhibit the region's lowest genetic diversity, emerging as isolated from all other regional populations across the deep‐water barrier of the Singapore Straits (Figures 1 and 3). To ensure optimal conservation of *C*. *rotundicauda* across the Singapore Straits, restoration of mangrove habitats may not be as cost‐effective as conserving extant mangrove habitats in shallow parts of the Straits. Habitat restoration should primarily focus on creating corridors to provide increased connectivity between isolated populations characterized by low diversity. In the immediate future, further loss of genetic diversity in the local *C*. *rotundicauda* population unfortunately appears inevitable—intense conservation efforts notwithstanding—so long as sea levels continue to rise or persist at the current level. As for *T*. *gigas*, rapid future sea‐level rise may facilitate a slight increment in the level of genetic diversity. However, the precise fate of local populations also depends on small‐scale habitat dynamics and human disturbance and development, all of which need to be taken into consideration by conservation planners (Di Nitto et al., 2014; Jiang et al., 2016).

Our results identified the southeastern end of the Straits of Malacca as a stronghold featuring the highest genetic diversity for both horseshoe crab species (Figure 1), so this area should be considered of especially high conservation value. High genetic diversity may reflect a high abundance of individuals (Frankham, 1996), and this—in turn—is possibly linked to relatively high levels of productivity as indicated by the regionally highest annual levels of chlorophyll *a* concentration in this area (Siswanto & Tanaka, 2014). Systematic in‐depth population surveys would provide invaluable additional data to evaluate each species’ conservation status for long‐term conservation planning.

Recent conservation concern for horseshoe crabs in the Singapore Straits has revolved around both species at equal measure, with suggestions that *T*. *gigas* may even be the more endangered species because of fewer detections (Cartwright‐Taylor, 2015; Davison et al., 2008). Our results demonstrate that *T*. *gigas*, with its panmictic population structure and strong recolonization potential, is of much lesser concern than the poorly dispersive *C*. *rotundicauda*, which suffers from a long‐term trend of decreasing effective population size throughout the Holocene. This dichotomy highlights the importance of studies such as ours in identifying the right conservation targets in our efforts to mitigate the effects of future climate change. While limited to horseshoe crabs, our study provides a blueprint for impending research to help safeguard the Earth's coastal and marine organismic communities.

## CONFLICT OF INTEREST

The authors declare no conflicts of interest.

## Supporting information

Supplementary Material

Supplementary Material

Supplementary Material

Fig S1‐S5

Table S1

Table S2

Table S3

Table S4

## Data Availability

The data that support the findings of this study are openly available in the Dryad digital repository at http://doi.org/10.5061/dryad.5mkkwh75h.
