# Supplementary material for: The different fates of two Asian horseshoe crab species with different dispersal abilities
Source: Evol Appl. 2021 Jul 23;14(8):2124–33. doi: 10.1111/eva.13271 (PMC8372080; doi:10.1111/eva.13271)
Supplement: Supplementary file 4 — Fig S1‐S5 [file EVA-14-2124-s005.docx]

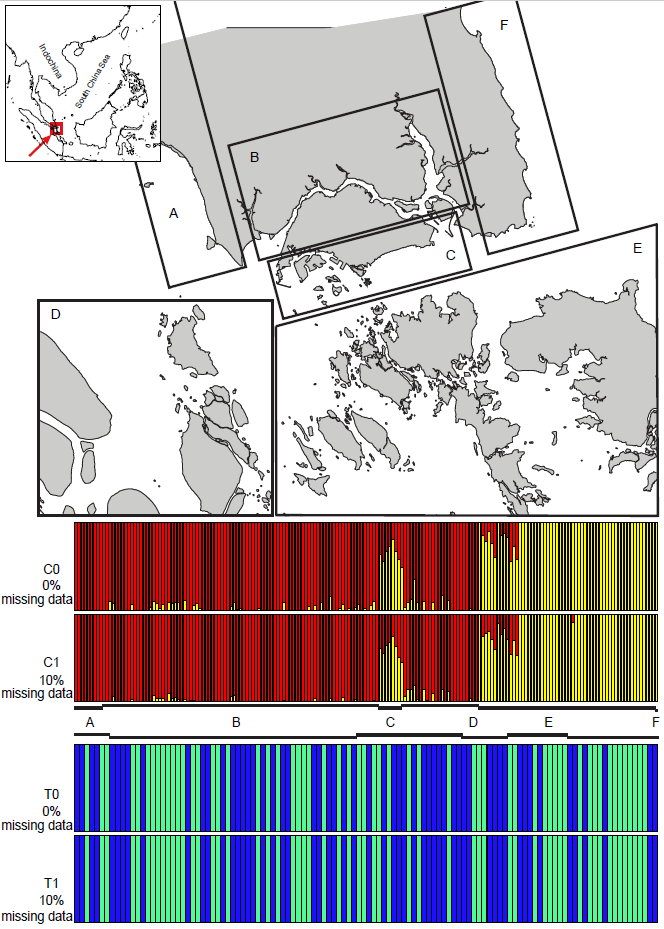


**Fig. S1. Genetic clustering using *ADMIXTURE* (*K* = 2) of two horseshoe crab species across the Singapore Straits.** Individuals are assigned to six regions as illustrated on the map: A) west coast of Malay Peninsula, B) Johor Straits, C) north of Singapore Straits, D) west of Singapore Straits, E) south of Singapore Straits, and F) east coast of Malay Peninsula. Bar plots illustrate assignment of individuals to two ancestral contributions, estimated across four SNP datasets (C0, C1, T0 and T1), respectively.

**
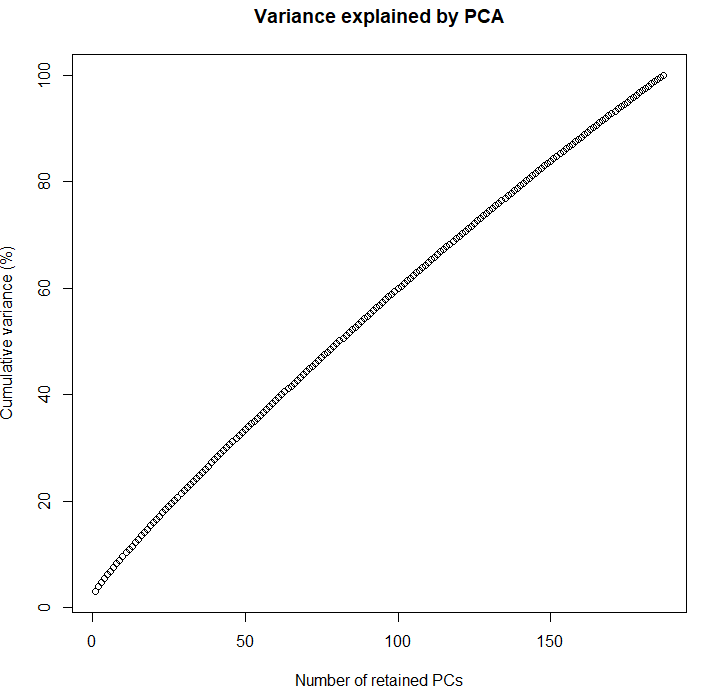

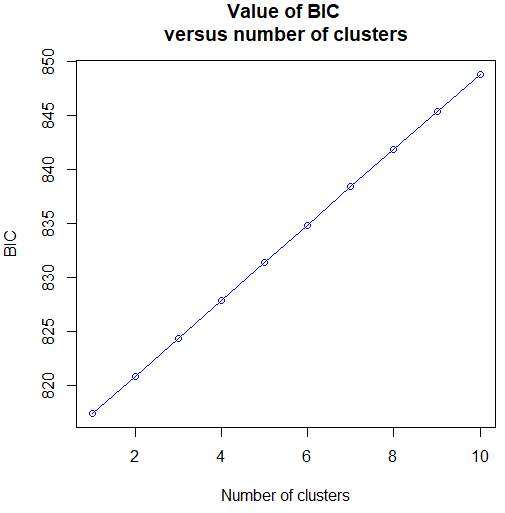

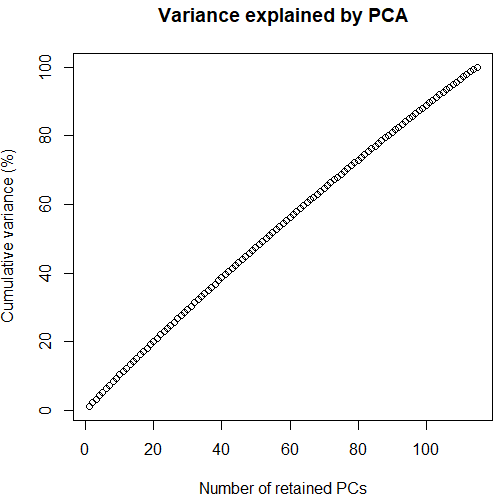

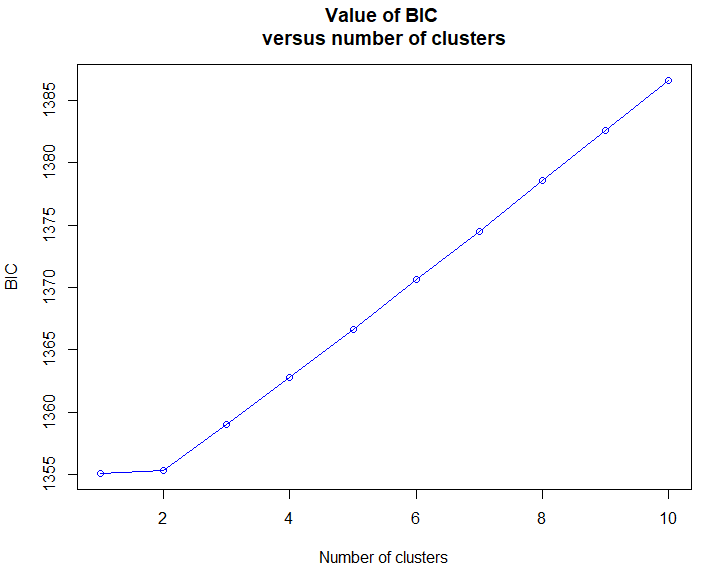
Fig. S2. Cumulated variance explained by the eigenvalues of the PCA and the BIC for the DAPC clustering.** Charts on top are for *C. rotundicauda* (dataset C0); charts on the bottom are for *T. gigas* (dataset T0)


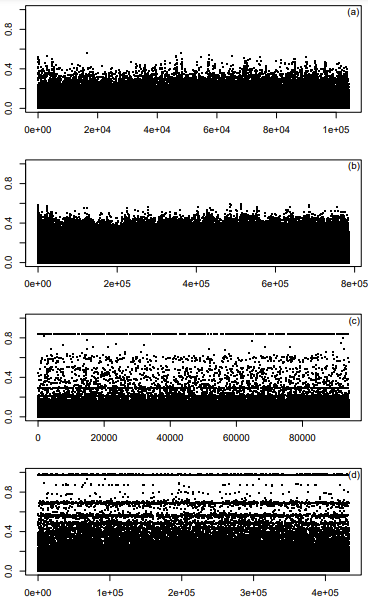
**Fig. S3. Correlations between the dominant principal component (PC1) and SNP genotypes.** (a) *C. rotundicauda* with no missing data (dataset C0); (b) *C. rotundicauda* with 10% missing data (dataset C1); (c) *T. gigas* with no missing data (dataset T0); (b) *T. gigas* with 10% missing data (dataset T1).


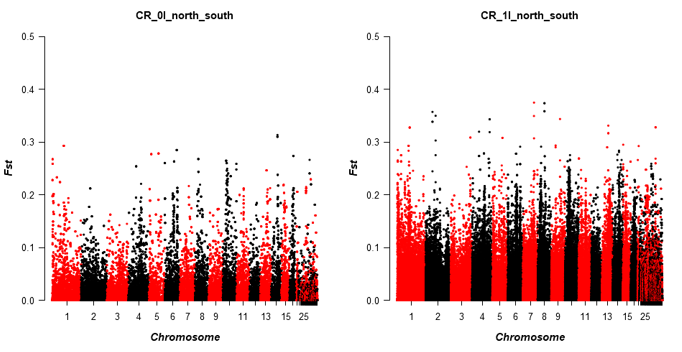
**Fig. S4. Manhattan plots showing genome-wide pairwise Fst values between non-admixed individuals from probable ancestral populations identified by *ADMIXTURE*.** Left: *C. rotundicauda* with no missing data (dataset C0). Right: *C. rotundicauda* with 10% missing data (dataset C1).


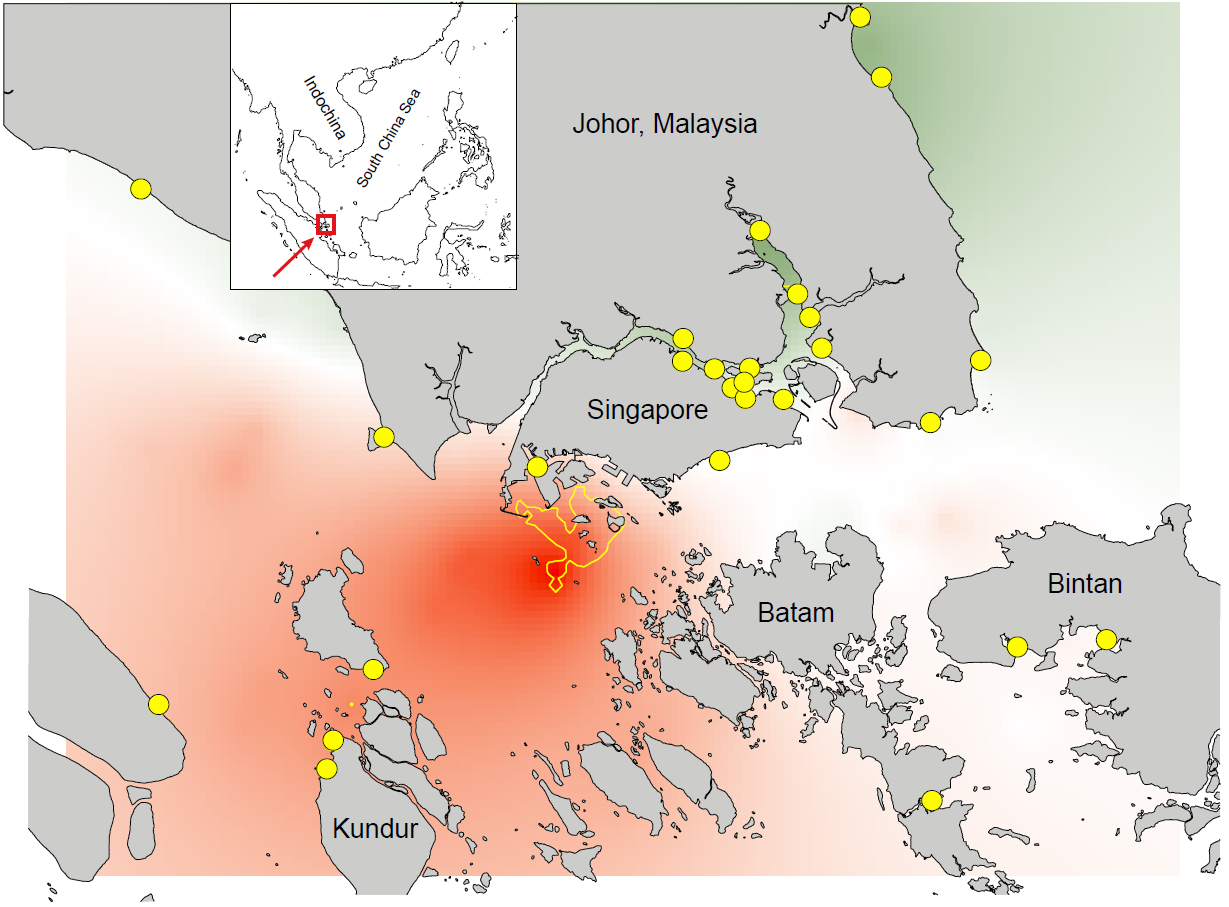


**Fig. S5. Estimated distribution of dispersal resistance to *T. gigas* across the Singapore Straits, generated using *DResD*.** Red indicates areas of high resistance whereas green indicates areas of low resistance; sampling localities are indicated with yellow dots; yellow contour lines enclose areas that have sufficient statistical power (> 0.8); there is insufficient statistical significance for resistance estimates across the entire study area.
